# Association between subclinical hypothyroidism and metabolic disorders: A retrospective chart review study in an emerging university hospital

**DOI:** 10.1002/jcla.22983

**Published:** 2019-07-19

**Authors:** Khaled Aldossari, Sameer Al‐Ghamdi, Jamaan Al‐Zahrani, Anwar Al Jammah, Bader Alanazi, Abdulilah Al‐Briek, Mohammad Alanazi

**Affiliations:** ^1^ Department of Family Medicine, College of Medicine Prince Sattam bin Abdulaziz University Alkharj Saudi Arabia; ^2^ Department of Endocrinology, College of Medicine King Saud University Riyadh Saudi Arabia; ^3^ Undergraduate Medical Student, College of Medicine Prince Sattam bin Abdulaziz University Alkharj Saudi Arabia

**Keywords:** Al Kharj city, gender, metabolic disorders, subclinical hypothyroidism, TSH

## Abstract

**Background:**

Subclinical hypothyroidism is defined as an increase in serum levels of Thyroid Stimulating Hormone (TSH) above the normal range, without alteration of total T4 concentrations that is reported to have association with various metabolic conditions. The study aimed to investigate any association between subclinical hypothyroidism and metabolic disorders in Al Kharj city.

**Methods:**

This is a cross‐sectional study that included review of patients’ charts from prince Sattam bin Abdul‐Aziz University, Al Kharj, Saudi Arabia, from August 1 to November 30, 2016. Data were analyzed with SPSS version 21. Descriptive statistics were obtained as frequencies. Pearson chi‐square analysis was used to assess any differences between disease status and study variables. *P*‐value < 0.05 was considered significant.

**Results:**

The mean age was 30.65 ± 13.3 with a female predominance. The average BMI was 29.5 ± 7.71; 46 (11.5%) had hypertension, 52 (46.8%) had diabetes, 173 (44%) had anemia, and 192 (56%) had vitamin D deficiency. Due to increased TSH levels, male gender had higher prevalence of subclinical hypothyroidism with *P*‐value < 0.001 and 0.011, respectively.

**Conclusion:**

Subclinical hypothyroidism is a significant topic worldwide whose prevalence is rising. In this study, we could not find any significant association between subclinical hypothyroidism and metabolic disorder. Further longitudinal studies with large sample size are needed to study this association.

## 1 INTRODUCTION

Subclinical hypothyroidism is defined as an increase in serum levels of TSH (Thyroid Stimulating Hormone) above the normal range, without alteration of total T4 concentrations or free T4. This biochemical profile may indicate the presence of mild hypothyroidism, with an increased potential risk of metabolic abnormalities and cardiovascular diseases seen in adults. The term subclinical denotes the presence of a disease without obvious symptoms, which means that evolution of the disease might be at an early stage. Subclinical hypothyroidism is classified into a mild subclinical hypothyroidism with TSH levels between 4.0 and 10.0 mIU/L and a severe subclinical hypothyroidism with TSH > 10.0 mIU/L. Subclinical hypothyroidism affects women more than men. Even though there is high risk in patients with severe subclinical hypothyroidism to develop into hypothyroidism, the risk is also great in patients with mild subclinical hypothyroidism.^1^

Numerous studies have been conducted to investigate the correlation between subclinical hypothyroidism, metabolic syndrome, and cardiovascular risk factors. Risk of clinical manifestations is 2‐3 times greater than individuals with normal TSH levels.^2^ In 2011, a study was carried out in Korea demonstrated that high normal TSH level is significantly associated with an increased prevalence of metabolic syndrome.^3^ In Japan, cross‐sectional and longitudinal follow‐up studies were published in 2013 observed strong correlation between subclinical hypothyroidism and metabolic syndrome.^4^ A study carried out in 2014 in Serbia revealed that traditional cardiovascular risk factors were more frequent in subclinical hypothyroidism patients compared with euthyroid patients.^5^ However, a systematic review that was done in 2015 in China showed that patients with type 2 DM are more likely to have subclinical hypothyroidism compared with healthy population.^6^

Significant risk factors for subclinical thyroid dysfunction in Nepal included family history of thyroid illness having relative risk (RR) of 2.57, smoking with RR of 2.56, and female gender with RR of 1.44.^7^ Various studies showed the association of its increased incidence with increasing age. In India, a population‐based study found high occurrence of subclinical hypothyroidism in women (11.4%) compared with men (6.2%). The same study found that increasing age has a significant association with it.^8^ Likewise, other studies also found increased prevalence of subclinical hypothyroidism with advancing age of women and those having higher BMI; however, a study conducted in Saudi Arabia did not find any significant variance between BMI groups. Moreover, unidentified thyroid dysfunction could create a negative impact on diabetes as well as its complications along with a high frequency of nephropathy and retinopathy in patients with diabetes having subclinical hypothyroidism.^9^ Therefore, managing subclinical hypothyroidism in patients with diabetes may prove extremely beneficial.

As far as the studies that were done in Saudi Arabia, we found a literature review on a cohort study done in 2015 revealed that the prevalence of subclinical hypothyroidism in heart failure patients was 14.4%,^6^ while it was 14.9% in pregnant women in a prospective study done in 2011.^10^ Another study from Saudi Arabia reported 28.5% of patients with type 2 diabetes have thyroid dysfunction,^9^ while a non‐significant trend was noted in TSH levels with high blood pressure and hyperlipidemia.^11^ Unfortunately, there are no studies done in Saudi Arabia or even in Arab countries to determine the correlation between subclinical hypothyroidism and metabolic diseases. The study aimed to investigate any association between subclinical hypothyroidism and metabolic disorders in Al Kharj city.

## 2 METHODOLOGY

The retrospective chart review was conducted in the university hospital of Prince Sattam bin Abdul‐Aziz University, Al Kharj, Saudi Arabia. A chart review for all the patients diagnosed with subclinical hypothyroidism having elevated levels of serum TSH associated with normal T4 who came to the outpatient clinic from September 1, 2015, to October 30, 2016, was done for the study. The study period was from August 1 to November 30, 2016.

Given that the prevalence of subclinical hypothyroidism in Saudi Arabia is 7% according to study done in Jeddah and the total population of Al kharj governorate is 376 325,^12^ by using Raosoft.com website, the sample size was calculated by keeping the confidence level at 95% and margin of error at 5%; the total sample was determined as 379, which was adjusted to 420 by keeping response rate in consideration. According to the World Health Organization, overweight was defined as a BMI greater than or equal to 25, whereas obesity was a BMI greater than or equal to 30.^13^ A trained research assistant constructed a chart review of patient files. We have taken TSH test to know whether the patient has subclinical hypothyroidism; the normal range of TSH was from 0.40 to 4.70 ulU/mL and T4 was from 12 to 22 Pmol/L according to the University Hospital Lab,^14^ so any patient with high TSH and normal T4 was diagnosed as subclinical hypothyroidism; we have taken the following tests to study metabolic disorders: levels of hemoglobin, ferritin, vitamin D, hemoglobin A1C, albumin, Alkaline phosphatase (ALP), Alanine transaminase (ALT), Aspartate aminotransferase (AST), creatinine, urea/blood urea nitrogen, High‐density lipoprotein (HDL), Low‐density lipoproteins (LDL), and triglyceride. The normal range for those tests is given in Table 1.

**Table 1:** Laboratory parameters and their values

Laboratory tests	Values
Hemoglobin	Male 14.0‐17.5 g/dL Female 12.3‐15.3 g/dL
Ferritin	12‐150 ng/mL
Vitamin D	20‐100 ng/mL
hemoglobin A1c	4.8%‐5.9%
Albumen	39‐58 g/L
ALP	35‐129 U/L
ALT	0‐41 U/L
AST	0‐40 U/L
Creatinine	62‐106 umol/L
Urea/BUN	2.10‐7.14 mmol/L
HDL	0.9‐1.68 mmol/L
LDL	2.59‐3.34 mmol/L
Triglycerides	0.5‐2.26 mg/dL

Patients from 2 to 70 years old were included in the study, whereas patients who were already diagnosed with hypothyroidism and were on the treatment, or any patient treated surgically for thyroid gland disorders, pregnant patients and admitted patients with other medical illnesses were not included in this study.

The research received ethical approval from Local Institutional Review Board. Confidentiality of patients’ data was ensured at all levels of the study.

Data entry and data analysis were carried out using Statistical Package for Social Science (SPSS) software version 21, which was developed by SPSS Inc, and acquired by IBM in 2009. Descriptive statistics that obtained as frequencies were used for continuous variables, whereas mean and standard deviation was used for categorical variables. A chi‐square test was applied to examine the association between different categorical variables. *P*‐values < 0.05 were considered statistically significant.

## 3 RESULTS

Table 2 shows that the total number of participants was 420. Their mean age was found to be 30.65 ± 13.3. Many participants (57.4%) lie in 16‐30 years of age group followed by 31‐45 years (24%). Majority of the respondents (84.5%) were females and 15.5% were males. Among these, 45.8% were obese, 28.5% were overweight, and 20.6% of people fell in healthy BMI group. However, the average BMI was 29.5 ± 7.71. Regarding the occurrence of diseases, 88.5% of individuals had no hypertension, whereas 11.5% had hypertension.

**Table 2:** Baseline characteristics of the study participants

Characteristics	N (%)
Age in years (N = 420)	
2‐15′	22 (5.2)
16‐30	241 (57.4)
31‐45	101 (24)
45‐60	40 (9.5)
60 to ≥70	15 (3.6)
Mean age: 30.65 ± 13.3	
Gender (N = 420)	
Female	355 (84.5)
Male	65 (15.5)
BMI (N = 420)	
Underweight	18 (5.1)
Healthy	73 (20.6)
Overweight	101 (28.5)
Obese	162 (45.8)
Mean BMI: 29.5 ± 7.71	
Hypertension (N = 400)	
Yes	46 (11.5)
No	354 (88.5)
Diabetes (N = 111)	
Yes	52 (46.8)
No	59 (53.2)
Anemia (N = 392)	
Yes	173 (44.1)
No	219 (55.9)
Vitamin D deficiency (342)	
Yes	192 (56.1)
No	150 (43.9)
Renal function	
Creatinine (N = 291)	
Normal	157 (51.1)
Abnormal	150 (48.9)
BUN (296)	
Normal	275 (92.9)
Abnormal	21 (7.1)
Total cholesterol (N = 268)	
Normal	196 (73.1)
Abnormal	72 (26.9)

Approximately half of the participants (46.8%) had diabetes, 44% had anemia, and 56% had vitamin D deficiency. The biochemical analysis on the renal function of patients showed that the levels of creatinine (51%) and BUN (93%) were within normal limits. Likewise, 73% had normal cholesterol levels. TSH levels were found high in 85.2% and were normal or low in 14.8% of the individuals, whereas T4 levels were abnormal in only 0.2% of the study population.

A subanalysis was conducted for the individuals who were 40 years of age or more as shown in Table 3. Overall, the sample contained 94 participants who belonged to this age group where the mean age was 50.77 ± 9.7. Among these, 4.4% were healthy, 20.6% were overweight, and 75% were obese with mean BMI of 35.36 ± 7.8. Regarding the presence of co‐morbidities, 28% had hypertension, 61% had diabetes, 38% had anemia, and 42.9% had vitamin D deficiency in this subgroup of individuals. For the renal function, the abnormal creatinine and BUN levels of the participants from this group were 47.2% and 7.6%, respectively. The cholesterol level of 7.6% of adults was not in the normal range.

**Table 3:** Baseline characteristics of the subgroup

Characteristics (N = 94)	N (%)
Mean age: (N = 94) 50.77 ± 9.7	
Gender (N = 94)	
Female	79 (84)
Male	15 (16)
BMI (N = 68)	
Underweight	0 (0)
Healthy	3 (4.4)
Overweight	14 (20.6)
Obese	51 (75)
Mean BMI: 35.36 ± 7.8	
Hypertension (N = 89)	
Yes	25 (28.1)
No	64 (71.9)
Diabetes (N = 54)	
Yes	33 (61.1)
No	21 (38.9)
Anemia (N = 89)	
Yes	34 (38.2)
No	55 (61.8)
Vitamin D Deficiency (N = 91)	
Yes	39 (42.9)
No	52 (57.1)
Renal function	
Creatinine (N = 89)	
Normal	47 (52.8)
Abnormal	42 (47.2)
BUN (N = 92)	
Normal	85 (92.4)
Abnormal	7 (7.6)
Total cholesterol (N = 93)	
Normal	60 (64.5)
Abnormal	33 (35.5)

## 4 DISCUSSION

The prevalence of subclinical hypothyroidism in our study was 85%. The present study aimed to observe any possible relationship between subclinical hypothyroidism and metabolic conditions among males and females across various age groups and all BMI categories but did not find any significant associations on logistic regression; however, TSH and gender were significant with chi‐square analysis. The mean age of the study participants was 30.65 ± 13.3 with a female predominance in the sample with majority of the participants lying in the age group of 16‐30 years (57.4%) and 31‐45 years (24%). High BMI was prominent in 45.8% and 28.5% of obese and overweight of the study population, respectively, with an average BMI of 29.5 ± 7.71. Only 11.5% had hypertension, 46.8% had diabetes, 44% had anemia, and 56% had vitamin D deficiency. Many of them had normal renal function and serum cholesterol. High TSH and normal T4 levels were found in majority of the participants.

Data showed that the prevalence of subclinical hypothyroidism in other Arab countries was 2.3% in Libya,^15^ while in Saudi Arabia, the newly diagnosed subclinical hypothyroidism was 11.1% in obstructive sleep apnea (OSA) patients and 4% in non‐OSA patients.^16^ In Korea, it was reported between 0.2% and 9.7% in adult population, generally about 2%‐3%.^17^ According to a review done by Al Shahrani, the prevalence of subclinical hypothyroidism in Arab region is comparatively low, which could possibly be the result of small sample size used for these studies.^18^ Another study from Jeddah having female predominance in the sample (82%) showed that 71.9% of the patients with clinically evident hypothyroidism had metabolic syndrome.^19^

In the western world, in the US adult population subclinical hypothyroidism was found to be around 0.7% being more common in women than in men.^20^ The significant association between TSH and gender in our study was also confirmed by various other studies, being evident in females.^7^, ^8^, ^9^ Subclinical hypothyroidism was also found to be associated with adverse cardiovascular consequences in women aged 55 years and older. Likewise, its simultaneous occurrence with Antithyroid antibodies may reflect ultimate progression to more severe form of hypothyroidism. A subgroup investigation on individuals aged 65 and more found that the maximum risk of cardiovascular or all‐cause mortality was found in this subgroup.^21^

In a cross‐sectional study, which was a population‐based Nord‐Trøndelag Health Study, the risk of mortality due to coronary heart disease was significantly higher in people with an average age of about 60.1.^22^ A considerable number of persons with subclinical hypothyroidism eventually develop overt hypothyroidism on a yearly rate of 4.3%‐8%, with a larger predisposition in the aged population^11^ On a similar note, Wickham survey revealed 10% of women aged 55‐64 years having higher TSH levels.^23^ This proposes that our study population might reasonably represent the general population. However, increasing the sample size might show any potential relationship between other factors under study with subclinical hypothyroidism.

Cai et al^24^ in a meta‐analysis indicated the association of subclinical hypothyroidism with increased systolic and diastolic blood pressures, whereas non‐significant upward trend was noticed in TSH levels with increasing blood pressure, BMI, and hyperlipidemia in the population of Riyadh, which was in accordance with our study results.^11^ Hajieh et al^25^ in a study from Saudi Arabia found 20% of patients with type 2 diabetes have clinical or subclinical hypothyroidism. It was evaluated to play a vital role in the development of metabolic syndrome, specifically in those patients who have insulin resistance and increased waist circumference which are recognized for having a direct negative effect on cardiovascular mortality as well as morbidity.^19^

## 5 CONCLUSION

Subclinical hypothyroidism is recognized greatly as a probable cardiovascular risk factor that might interfere with total morbidity and mortality. Data supporting such findings as well as any other adverse clinical outcomes or treatment benefits are also limited. Since subclinical hypothyroidism is associated with augmented cardiovascular risk, a systematic review indicated that benefits of thyroid hormone replacement are reported to avoid its progression to overt hypothyroidism and specifically when the focus is to improve lipid profile, reduce LDL, and overall better cardiac function. Therefore, a cost‐effective strategy of screening the patients for thyroid dysfunction can be a favorable strategy.

## 6 STRENGTH AND LIMITATIONS

The published literature from Saudi Arabia lacks much information regarding subclinical hypothyroidism and its clinical and metabolic affiliations. Our study added some more information into the existing data about this topic. Another strength of this study is the use of WHO‐approved definitions and laboratory sample cutoffs. However, this study is a cross‐sectional study and so causal inferences were not achieved. Secondly, the proportion of women was higher in the sample so the real gender distribution could not be appreciated. Almost all the participants had subclinical hypothyroidism, there was no control group, that is the comparison group, and association was not appropriately evaluated with small sample size.

## 7 RECOMMENDATIONS

It is essential to carry out clinical trials in order to generate newer insights to guide clinical practitioners to screen, diagnose, and treat all the suspected cases of subclinical hypothyroidism. Additionally, population‐level screening as a part of a routine workup should also be considered. It is equally important to revise the normal reference ranges for TSH according to the population and a standardized value to be followed in order to ensure that the results are reproducible and accurate and so unnecessary usage of thyroid hormones would be prevented.

## CONFLICT OF INTEREST

The authors declare that they have no competing interests.

## AUTHORS' CONTRIBUTIONS

This work was performed as collaboration among all the authors. KAD and SAG participated in the study design and wrote the first draft of the manuscript. JAZ and AAJ collected and processed the samples. BAA, AAB, and MAA participated in the study design and performed the statistical analyses. All of the authors read and approved the final manuscript.

## ETHICAL APPROVAL

The study was approved by the Ethics Committee of Prince Sattam bin Abdulaziz University Institutional Review Board.

## DATA AVAILABILITY

Data are available upon request from the authors.

## CONSENT FOR PUBLICATION

Not applicable.
